# CT findings of a large primary pulmonary myxoid sarcoma: A case report

**DOI:** 10.1016/j.radcr.2022.06.029

**Published:** 2022-07-13

**Authors:** Silvi Yuliana, Fierly Hayati

**Affiliations:** a Radiology Resident, Department of Radiology, Faculty of Medicine Universitas Airlangga, Dr. Soetomo General Academic Hospital, Surabaya, Indonesia; b Thoracic Radiologist, Department of Radiology, Faculty of Medicine Universitas Airlangga, Dr. Soetomo General Academic Hospital, Surabaya, Indonesia

**Keywords:** Primary pulmonary myxoid sarcoma, Lung tumor, Lung mass

## Abstract

Primary pulmonary myxoid sarcoma (PPMS) is an extremely rare malignant mesenchymal tumor of the lung, with only less than 40 cases reported. We described a case of a 64-years-old man with a mass on the medial and lower lobe of the right lung confirmed as a primary pulmonary myxoid sarcoma on biopsy. Diagnosis of this tumor remains challenging because of its nonspecific clinical and imaging characteristics. This study emphasizes CT finding to improve the understanding of PPMS.

## Introduction

Primary pulmonary myxoid sarcoma (PPMS) is an unusual entity of malignant mesenchymal lung tumor [1], [2], [3], with only 37 cases reported until 2021 [1]. The first reported case of this tumor was presented in 1999 by Nicholson et al. [4]. At that time, it was still unclear about the malignant potential of this tumor entity. In 2011, Thway et al. reported a more significant number of patients with similar morphology, proven this tumor's malignant property, and termed this mass primary pulmonary myxoid sarcoma [5]. WHO classified this tumor as a mesenchymal lung tumor in 2015 [6].

The clinical symptoms and imaging findings of this tumor were non-specific, making a preoperative diagnosis of this tumor quite challenging [1,3]. A few pieces of literature have described the imaging finding of this tumor entity. We report one case of primary pulmonary myxoid sarcoma of the lung, emphasizing CT finding to improve the understanding of PPMS.

## Case report

A 64 years old male presented to our center with 2 weeks of coughing prior to hospitalization. He also complained of the right chest heaviness for 2 months and had a history of bloody cough one year before, with exertional dyspnea, anorexia, weight loss, and no fever. The patient was a heavy smoker and was able to consume roughly one pack a day. No history or family account of cancer, diabetes, hypertension, or other pulmonary infection was recorded.

On physical examination, his vital sign was normal with a blood pressure of 120/80 mmHg, heart rate of 90 x/m, respiratory rate of 22 x/m, body temperature of 36,4°C, and oxygen saturation of 98% in the room air. A slower movement of the right chest was noted at the chest examination, with dull percussion and a decrease in pulmonary sound in the right lower hemithorax. Another physical examination was within the normal limit. The laboratory result showed no significant abnormality and no increase in any tumor marker.

**Fig. 1:**
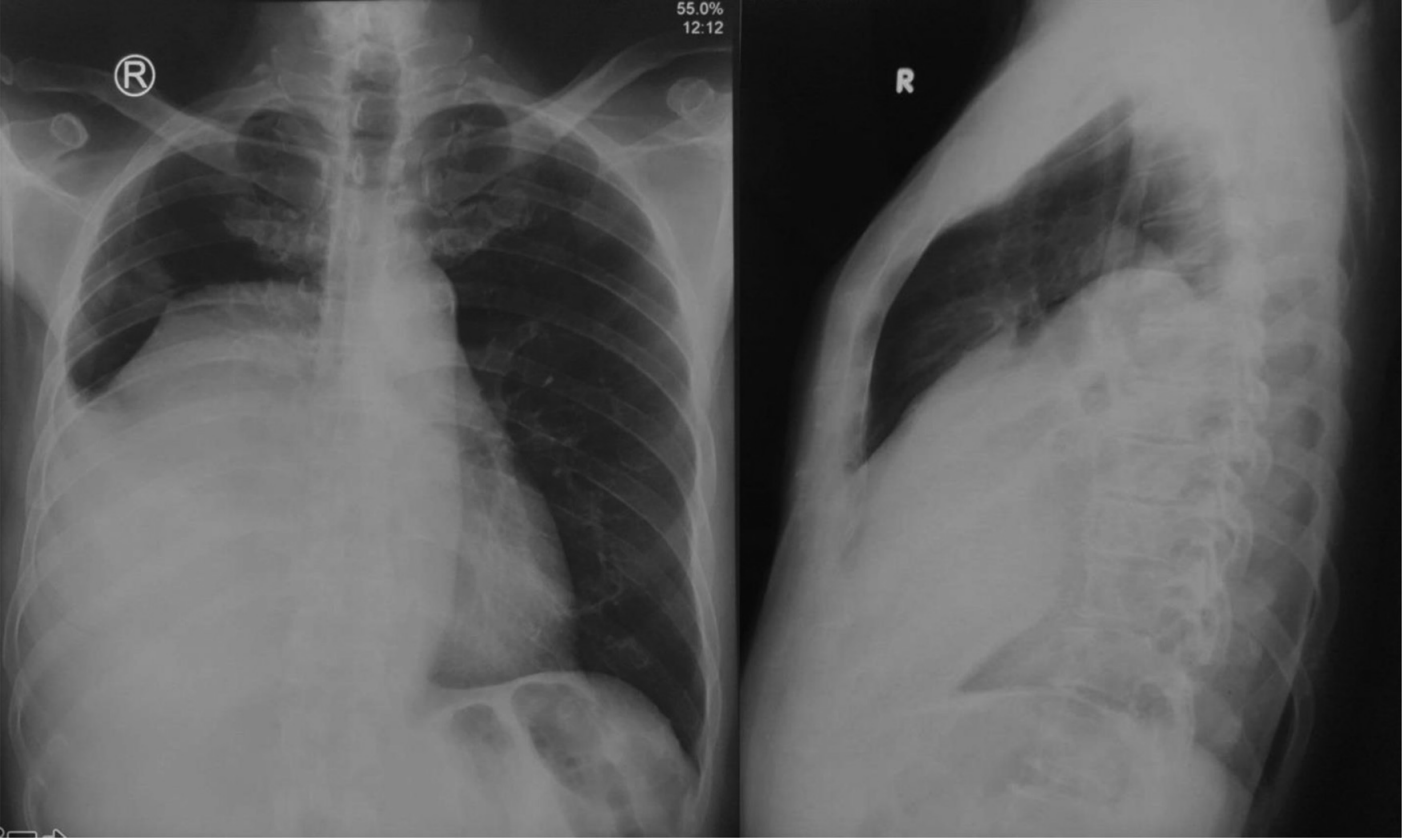
A chest x-ray AP/ Lateral projection showed large opacity with a defined border and regular margin in the right hemithorax covering the right hemidiaphragm and right anterior and posterior costophrenic angle, resembling a pleural effusion.

The plain radiograph of the chest showed an opacity on the right hemithorax obscuring the right hemidiaphragm and right anterior and posterior costophrenic angle, resembling a pleural effusion (Fig. 1).

**Fig. 2:**
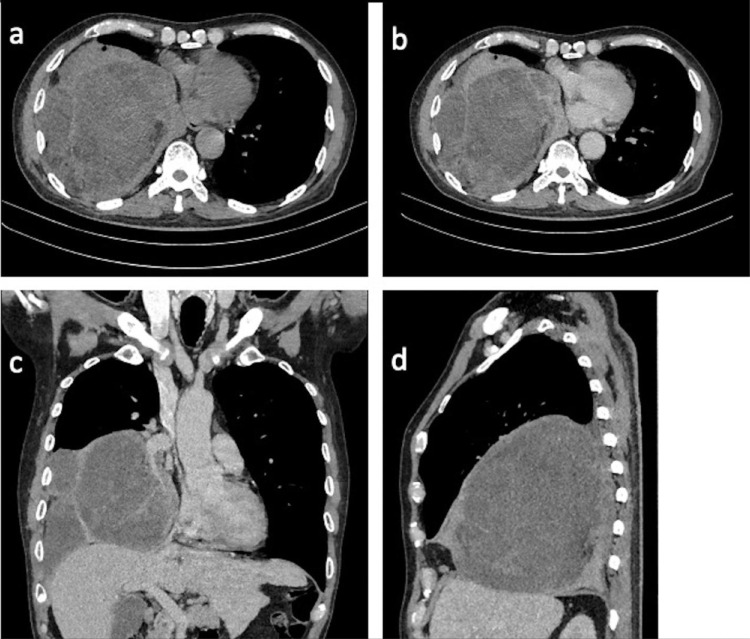
Chest CT, mediastinal window, unenhanced (a) and enhanced axial (b), coronal (c), and sagittal view (d). A solid mass (41 HU), measuring approximately 13.2 × 15.0 × 12.2 cm with fat and cystic component, clear margin, loculated, septated, with 4 mm septal thickness at the medial and lower lobe of the right lung (a). Contrast administration, mediastinal window, axial, coronal and sagittal view (b–d) showed contrast enhancement on solid and septated components (87 HU).

This finding was confirmed by CT scan, depicting a solid mass (41 HU), measuring approximately 13.2 × 15.0 × 12.2 cm with fat and cystic component, distinct margin, loculated, septated with 4 mm septal thickness, at the medial and lower lobe of the right lung (Fig. 2a, c, d). Moreover, a contrast-enhanced CT scan evaluation showed contrast enhancement on solid and septal components (87 HU) (Fig. 2b, c, d).

**Fig. 3:**
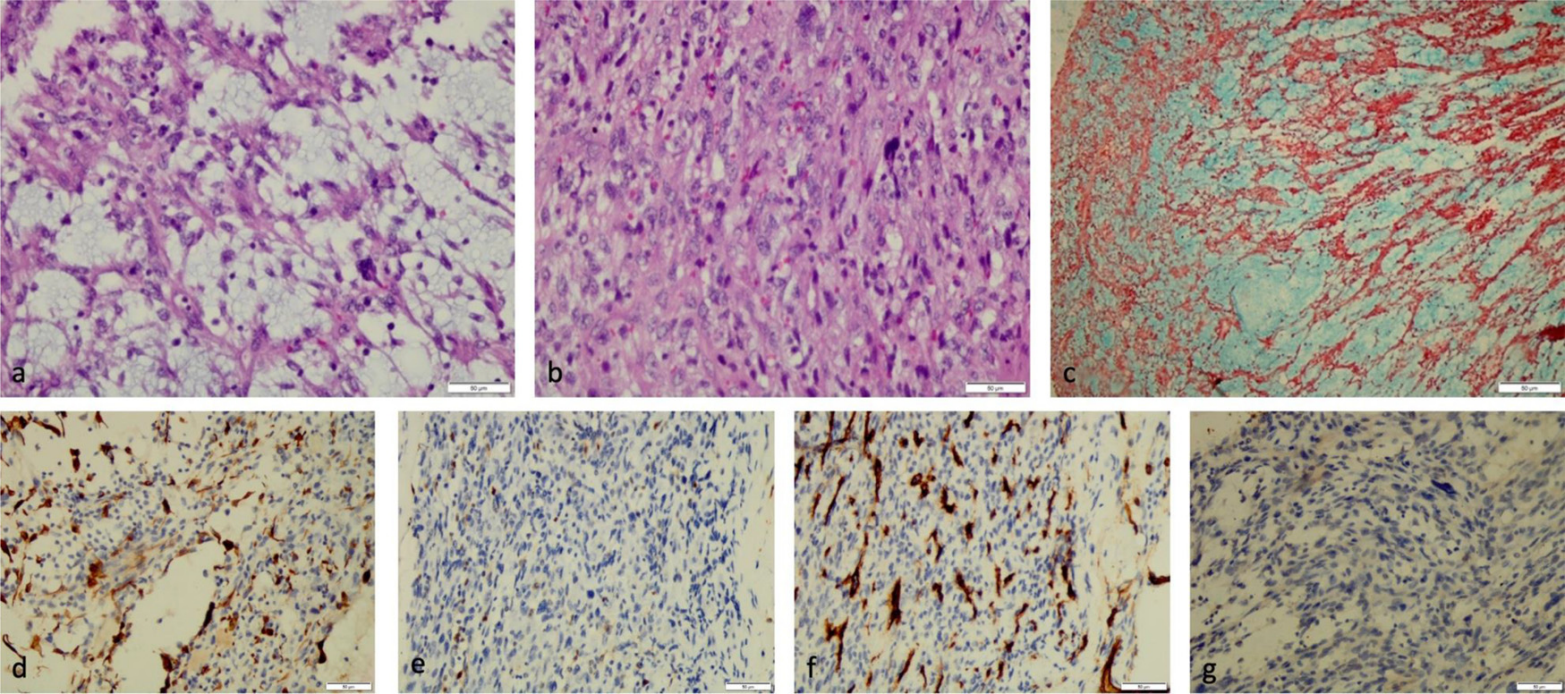
Pathology examination of the lung parenchyma showed a reticular arrangement of the tumor growth in the myxoid stroma, partially in the lobules, including the anaplastic cell proliferation with a round oval-spindle nucleus (a, ×400 magnification, H&E stain) with a mitotic rate of >20/10 HPF (b, ×400 magnification, H&E stain), and positive alcian blue stain (c, ×100 magnification). Immunostaining expressed a focally desmin-positive (d, ×400 magnification) and negative S 100 (e, ×400 magnification), CD 34 (f, ×400 magnification), and CK (g, ×400 magnification).

Afterwards, a bone survey, abdominal ultrasonography, and brain MRI were conducted to investigate the metastatic processes; nevertheless, no metastatic process was discovered. A subsequent open biopsy was performed, resulting in primary pulmonary myxoid sarcoma, confirmed by immunohistochemical staining (Fig. 3). The patient was then planned for surgical resection; however, his condition deteriorated, and the patient died due to sepsis several days after the biopsy result was obtained.

## Discussion

The prevalence of PPMS was comparable between female and male patients, with 19 females out of 38 cases reported [1,7]. The median age of the patient with PPMS was 44 years old, ranging from 23 to 80 years old [1].

Patients with this tumor exhibited non-specific signs and symptoms, with several asymptomatic cases discovered upon medical screening [1,7]. Others presented with productive or unproductive cough, hemoptysis, dyspnea, and weight loss [1,7]. In our case, the patient was introduced with a cough, right chest heaviness, a history of bloody cough, exertional dyspnea, anorexia, and weight loss.

PPMS is a tumor originating from mesenchymal elements of the bronchial wall, vessels, or pulmonary stroma [6,8]. This tumor typically arises in the airways, with varying locations and sizes [1,3,6]. It is often found in lung parenchyma with or without endobronchial involvement [3,6]. However, Kim et al. described that this tumor might grow outside the lung parenchyma, developing in an interlobar fissure [9].

The imaging feature of this tumor is uncertain. It frequently occurs in the right lung and is closely related to the bronchus [1]. The tumor has been reported to differ in measurement from nodular size to a large mass up to about 14 cm in diameter [1]. Furthermore, CT scans with contrast usually pictured these lesions as a mildly and heterogeneously enhanced mass [3,9,10]. In our case, the mass resided in the medial and lower lobe of the right lung, adjacent to the branch of the right bronchus, with an estimated size of 13.2 × 15.0 × 12.2 cm and contrast enhancement on the solid and septated components.

Microscopically, this tumor comprises lobules of delicate, lace-like strands and cords of lightly atypical round and spindle cells within a prominent myxoid stroma [6]. The mitotic rate of this tumor is up to 32 mitoses per 2 mm with atypical forms, although a high proportion of cases recorded 5 mitoses per 2 mm [6]. In addition, necrosis is seen in about 50% of these tumors and tends to be focal. On pathology examination, the myxoid stroma was alcian-blue positive [6].

No immunohistochemical markers support the diagnosis of this tumor [7,11], although the majority express vimentin [6,11]. Nevertheless, other common markers are negative, particularly cytokeratins, S 100, smooth muscle actin, desmin, CD34, and neuroendocrine [6]. In our patient, immunostaining examination revealed focally desmin-positive, a rare condition with only one case reported by Smith et al., strongly positive for desmin [12].

Most patients with PPMS require surgical treatments with satisfactory clinical outcomes [1]. Surgical excision available includes wedge resection, segment resection, lobectomy, and pneumonectomy, depending on the size of the tumor [1]. Fortunately, this neoplasm is classified as a low-grade malignancy; nonetheless, close follow-up is indispensable to detect further metastases or recurrence [1,3,7]. About 5 out of 39 reported patients with PPMS were found to have metastasis [1,7]. A good prognosis followed the treatment of PPMS, with only one death in a patient with brain metastases after surgery [1,5]. There was no evidence of recurrence of this tumor after surgical excision [1,7].

## Conclusion

Our findings on the PPMS case were in line with the previous findings that this is a rare condition with non-specific clinical presentations and CT characteristics. Although the definite diagnosis of primary pulmonary myxoid sarcoma could only be made after pathology examination, CT features remain helpful in specifying the nature, location, and relationship between the tumor and other structures. Most PPMS patients were treated surgically and had an excellent prognosis.
